# Isolation and identification of an AKAV strain in dairy cattle in China

**DOI:** 10.3389/fvets.2025.1574667

**Published:** 2025-05-20

**Authors:** Miaomiao Zhang, Menghua Deng, Sisi Zhao, Dengshuai Zhao, Yajie Zheng, Limei Qin, Han Gao, Mengmeng Zhao, Keshan Zhang

**Affiliations:** Guangdong Provincial Key Laboratory of Animal Molecular Design and Precise Breeding, School of Animal Science and Technology, Foshan University, Foshan, China

**Keywords:** Akabane virus (AKAV), bovine abortion, molecular detection, phylogenetic analysis, transmission electron microscopy (TEM)

## Abstract

Akabane disease is an arthropod-borne disease caused by Akabane virus (AKAV), which is characterized by abortion, premature birth, stillbirth, congenital arthrosis, and hydrocephalic anencephalic syndrome in pregnant cattle and sheep. The occurrence of AKAV was proved by RT-PCR amplification based on AKAV S fragment, virus isolation, cells inoculation, cytopathy, transmission electron microscopy, and gene sequencing. The PCR amplicon was approximately 850 bp and was sequenced, and molecular identification of AKAV was conducted through phylogenetic analysis of S gene sequence. The results indicated that AKAV isolated from cattle in this study was genetically close to the strain isolated from *Rhizomys pruinosus* in China in 2016. However, the outbreak in bamboo rats may have been a sporadic event. The probability that Akabane virus (AKAV) can spread in rodents and mammals is still uncertain and requires further investigation. Using transmission electron microscopy (TEM), AKAV particles displayed the typical morphology associated with bunyaviruses reported previously. In brief, the AKAV infection in cattle has been confirmed. This case report highlights the necessity for enhanced surveillance and preventive measures to mitigate the potential impact on livestock health and productivity.

## Introduction

Akabane virus is a member of the genus *Orthobunyavirus* and family *Bunyaviridae* and belongs to the Simbu serogroup in serological terms (1). The main clinical symptoms caused by AKAV were abortion in pregnant cattle and sheep, premature birth, stillbirth, congenital arthrosis, and hydrocephalic anencephalic syndrome in newborn calves (2, 3). AKAV mainly infects ruminants such as cattle, goats, and sheep and is prevalent worldwide. Akabane disease is a seasonal reproductive infectious disease (4, 5), which may cause large-scale spread through the bites of blood-sucking insects such as midges and mosquitoes. Several studies have discovered AKAV in arthropod, indicating arthropods played an important role in AKAV transmission (6–10). Therefore, AKAV poses a significantly potential risk of infection to ruminants, leading to seriously economic losses and more attention should be paid.

The outbreak of Akabane disease can cause great economic losses to the breeding industry which depend on prevalence, incidence rates, and economic modeling (11–13). In addition to transient viremia, infection of non-pregnant cattle with Akabane virus is typically asymptomatic, rendering detection challenging. Akabane disease can be confirmed by the combination of clinical diagnosis and laboratory detection. In clinical diagnosis, this disease should be differentiated from bovine viral diarrhea (BVD), Schmallenberg (SB), and Bluetongue (BT) (14, 15). Currently, the laboratory identification of Akabane virus is primarily divided into three methodologies: etiological analysis, immunological assays, and molecular biology techniques. Virus isolation and identification in etiological examination is the gold standard for the diagnosis of the disease, but it is difficult to popularize because of its high requirements on the operating environment, operators, expensive, and time-consuming. Immunological examination is mainly used in epidemiological investigation because it is simple and quick (16, 17). Molecular biology techniques such as polymerase chain reaction (PCR), commonly used in laboratories, are now relatively mature (7, 12).

AKAV is widely prevalent in most regions except Europe and America, epidemics of the disease have been reported in many countries (13, 18–24), and its prevalence is characterized by obvious seasonality, regionality, and cyclicity (6, 10). In recent years, AKAV has been reported in many regions of China (3, 13, 25–27), and current genotyping data predominantly classified Chinese AKAV strains as genogroup I (6, 26, 27). Like other *Orthobunyavirus*, AKAV is a single-stranded negative-sense, segmented RNA virus with a capsule, consisting of three segments (L, M, and S) (28). Based on the S gene sequence, AKAV can be divided into four genogroups (I–IV), genogroup I includes two subgenogroups, namely, Ia and Ib (29). Recent studies highlight divergent neuropathogenic mechanisms in Akabane virus (AKAV) genogroups. Genogroup II requires NSs protein for neuropathogenicity, while genogroup I exhibits NSs-independent virulence (30). Genogroup II shows heightened replication and cytopathic effects in bovine brain cells (31), linked to congenital brain lesions, whereas genogroup I (Iriki strain) demonstrates pronounced murine neuroinvasiveness absent in genogroup II (OBE-1 strain), underscoring genogroup-dependent virulence and tissue tropism (32).

In the current study, an AKAV infection in pregnant cow in China was reported. Through clinical observation, PCR appraisal, virus isolation, genetic evolution analysis, and TEM, the detection of AKAV in China has been confirmed. The nucleotide sequence obtained in this study has been deposited in the GenBank database under the accession numbers PQ567126-PQ567128.

## Materials and methods

### Ethics approval

Animal blood samples were collected after obtaining appropriate permission from the cattle farm owners.

### Virus sample

This AKAV infection case happened in a cattle farm in China. In April 2024, miscarriages were found in number of pregnant cattle and the aborted fetuses were deformed, and there was no response to antibiotic treatment.

### Cells and viral isolation

Vero cells (African green monkey kidney cells) and MDBK cells (Madin-Darby Bovine Kidney cells) were cultured in Dulbecco’s modified Eagle’s medium (DMEM, Gibco, United States) containing 1% antibiotic/antimycotic solution (Gibco, USA) and 10% fetal bovine serum (FBS, Shanghai XP Biomed, China). The cell lines were maintained at 37°C with 5% CO_2_.

The sample from sick cattle with typical clinical symptoms was selected. A comprehensive autopsy was performed, and the macro-pathological changes were recorded in detail. The pathological blood was removed from the typical lesion site carefully and then stored in a 50 mL centrifuge tube containing anticoagulant. Blood samples were centrifuged at 4°C, 1000 rpm for 5 min, and the supernatant was discarded. The pellet was washed with PBS, lysed with red blood cell lysis buffer at 25°C for 2 min, and centrifuged at 4°C, 500 rpm for 3 min. The cell pellet was resuspended in five times its volume with PBS, centrifuged again, and washed once more. Finally, the cells were lysed with 500 μL sterile water, and 200 μL lysate was inoculated onto confluent Vero cells and adsorbed for 2 h and subjected to passages. The viral supernatant from cells with obvious CPE was harvested. The viral liquid from cells with evident lesions was inoculated onto MDBK cells.

### RNA extraction and polymerase chain reaction

Viral RNA was extracted and reverse-transcribed into cDNA. RNA was extracted with TRIzol (Biosharp, China) and reverse-transcribed into cDNA using 5 × Script Prime RT Master Mix according to the manufacturer’s instructions. To identify the pathogen, primers were designed for reverse transcription-polymerase chain reaction (RT-PCR) based on the relatively conserved S gene fragment in the AKAV genome, AKAV-Forward: 5’-AGTAGTGAAC TCCACTATTAACTACGC-3′, AKAV-Reverse: 5’-AGTAGTGTGCT CCACTAATTAACTATAAA-3′ (33), and primers were synthesized by Sangon Biotech Biological Company (Shanghai, China). A 25 μL PCR mixture was used for each PCR amplification, which contained 12.5 μL of 2 × Magic Green Taq SuperMix, 1 μL of each primer (forward and reverse), and 500 ng cDNA and the remainder was supplemented with water. The PCR protocol included an initial denaturation at 95°C for 3 min, followed by 35 cycles of denaturation at 95°C for 15 s, annealing at 51°C for 15 s, and extension at 72°C for 15 s, with a final extension at 72°C for 5 min. Total RNA harvested from MDBK cells with evident lesions was extracted and reverse-transcribed, followed by RT-PCR detection, and RNA samples were sent to the Shanghai Tanpu Biotechnology Company (Sequencing Platform and Strategy: Illumina NovaSeq PE150) for whole-genome sequencing.

### Electron microscopy

For the visualization of AKAV particles by transmission electron microscopy (TEM) (34), samples containing virus were sent to Servicebio Biotech Biological Company (Wuhan, China). Micrographs were taken at appropriate magnifications to capture the morphology of the virus particles.

### Phylogenetic analyses

The amplified sequences were compared with those in the GenBank database using BLAST online program. Clustal W method was used to analyze the sequence consistency of nucleotides and amino acids. The phylogenetic tree was constructed by neighbor-joining method using MEGA version 7.0,^1^ 1,000 bootstrap repetitions, Kimura 2-parameter model, uniform rates, and pairwise deletion (35, 36).

## Results

### Molecular detection and identification of AKAV

**Figure 1:**
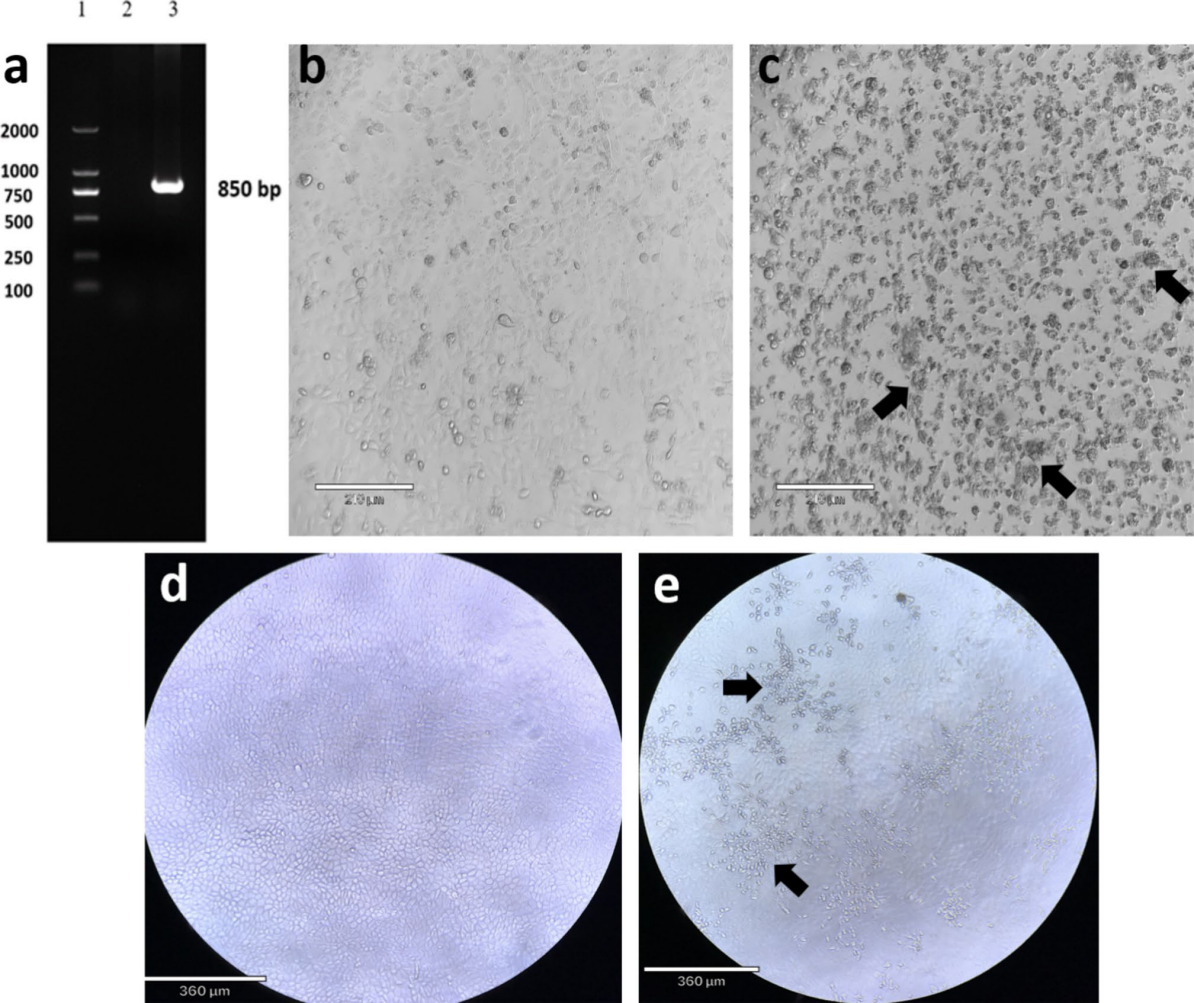
Gel electrophoresis RT-PCR assay for Akabane virus of S segment and cytopathic effect (CPE) of isolated Akabane virus-infected Vero cells. **(a)** Lane 1: DNA ladder; Lane 2: negative control; Lane 3: PCR product of S segment (850 bp). **(b)** Uninfected Vero cells (scale bar: 210 μm). **(c)** The isolated virus infected Vero cells, showing cell aggregation and detachment (scale bar: 210 μm). **(d)** Uninfected MDBK cells (scale bar: 360 μm). **(e)** The isolated virus infected MDBK cells, showing cells detachment (scale bar: 360 μm).

In this study, the pregnant cattle were aborted, the limbs of the aborted fetus were curved into a crouching shape, and the spine and head were deformed. Based on the clinical symptoms and pathological changes of the infected cattle, AKAV was preliminarily confirmed as the pathogen. Further confirmation was conducted through laboratory identification. The expected PCR fragment was obtained, the product length of the S fragment was approximately 850 bp, and there was no fragment in the negative control (Figure 1a). The PCR products obtained were sent to Sangon Biotech Biological Company (Shanghai, China) for sequencing to obtain more information about the virus. The result showed that the length of the S fragment was 838 bp. Whole-genome sequencing revealed that the S segment is 836 base pairs (bp) in length, with an A + T content of 54.68% and encodes 274 amino acids. The M segment is 4,281 bp in length, with an A + T content of 62.77% and encodes 1,424 amino acids. The L segment is 6,904 bp in length, with an A + T content of 64.81% and encodes 2,193 amino acids. The obtained sequences have been uploaded in the NCBI GenBank database, and PQ567126-PQ567128 were the accession numbers. In order to further confirm the presence of AKAV, viruses were isolated from blood tissue samples and inoculated onto Vero cells for blind passage. Obvious cytopathic effects (CPE) were observed at 48 h post-inoculation in the third blind passage generation. It was observed under the microscope that infected cells showed some pathological phenomena such as round contraction, aggregation, and shedding (Figure 1c). While uninfected Vero cells showed no apparent morphological changes or pathological alterations (Figure 1b). The RT-PCR result showed a target fragment of approximately 850 bp, consistent with the expected result. After the FS202301 virus isolate was inoculated into MDBK cells, CPE also occurred (Figure 1e) and no apparent morphological phenomena occurred on uninfected MDBK cells (Figure 1d).

### Electron microscopy observations

**Figure 2:**
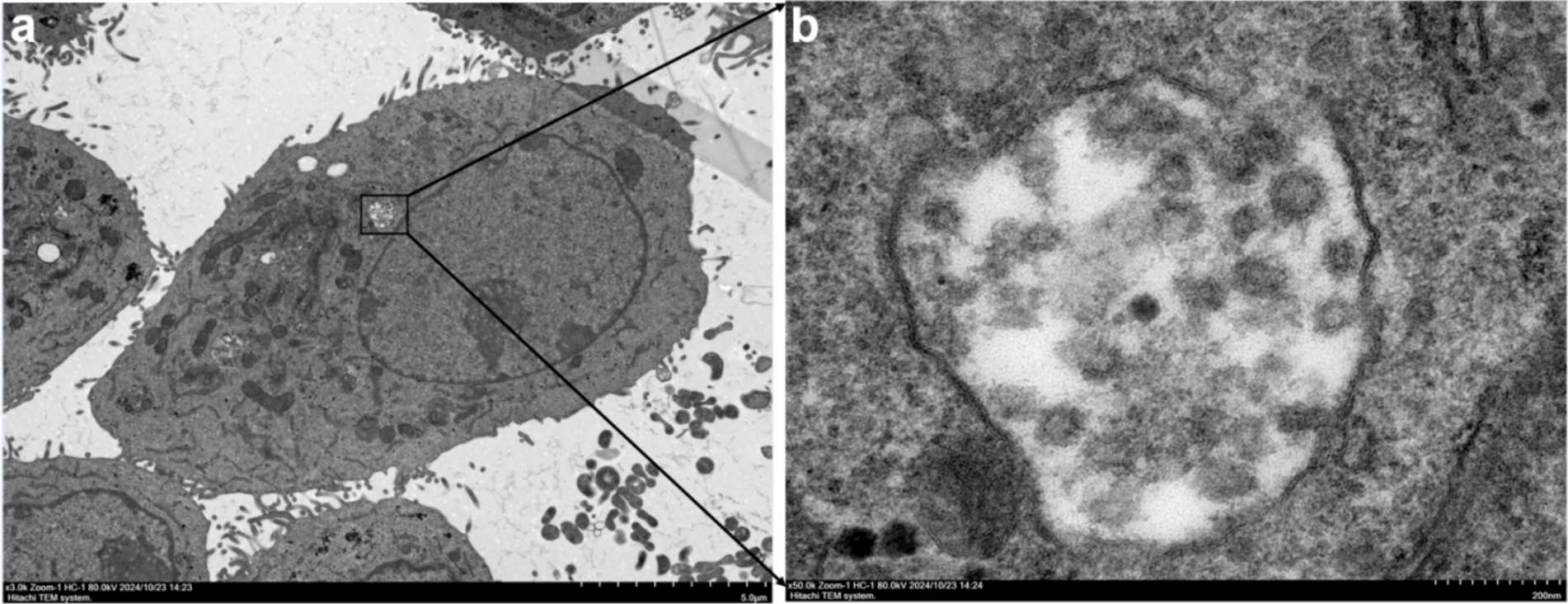
Transmission electron microscopy (TEM) images of AKAV particles. **(a)** Low magnification view (scale bar: 5.0 μm) of AKAV particles within an infected cell, highlighting the intracellular location of the virus. The black box indicates the area magnified in panel **a**. **(b)** High magnification view (scale bar: 200 nm) of AKAV particles, showing the characteristic morphology of the virus. The particles exhibit a spherical shape with some pleomorphic features, as observed in the samples.

Electron microscopy of the samples revealed the presence of spherical virus particles with a diameter of approximately 80–100 nm, consistent with the size of virus particles of the family *Bunyaviridae* reported in the literature (34, 36–39). The particles exhibited a typical morphology for bunyaviruses, with some showing a pleomorphic appearance. The micrographs provided clear evidence of viral particles in the samples, further confirming the presence of AKAV in the infected cattle (Figure 2).

### Phylogenetic analysis of the S gene of AKAV strain

**Figure 3:**
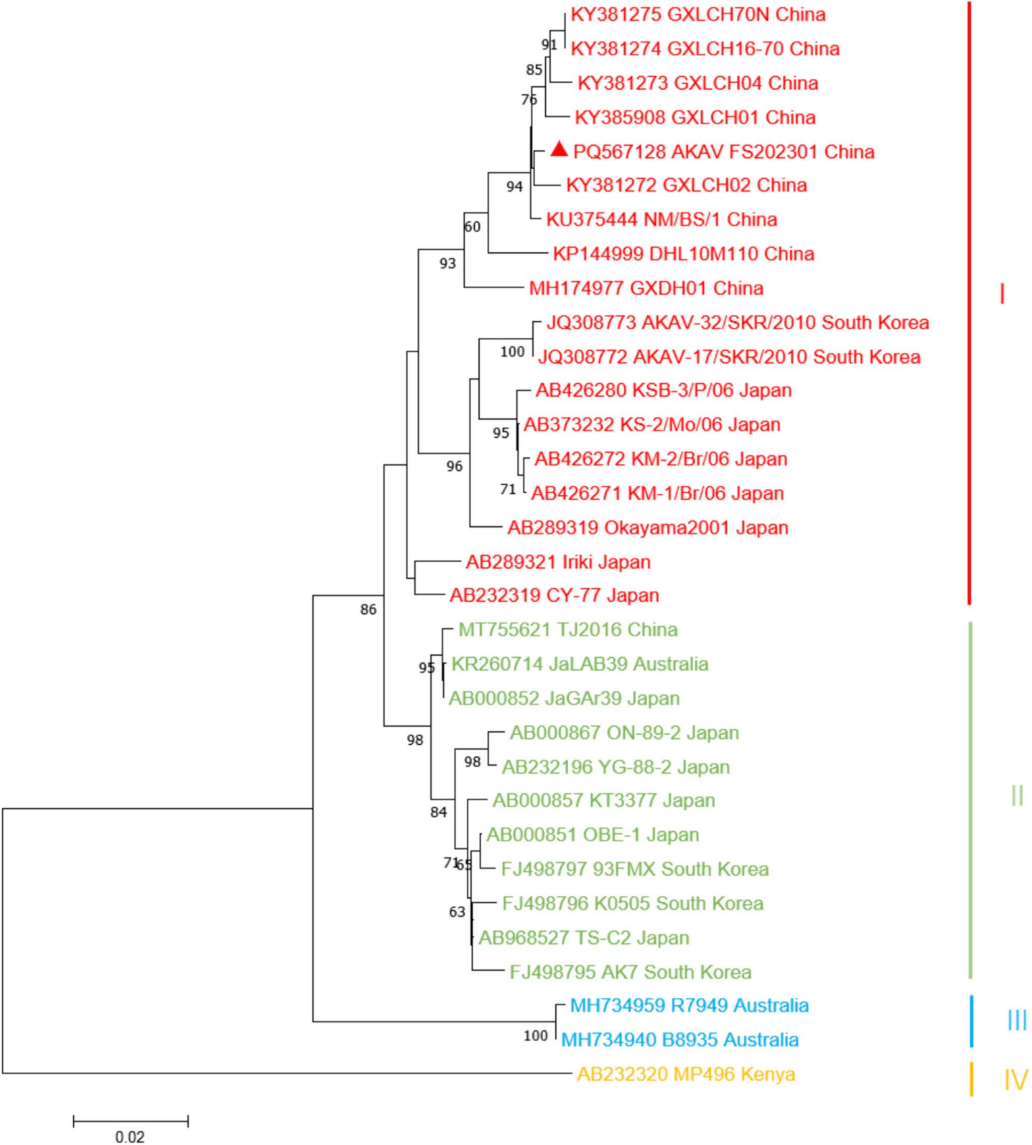
Phylogenetic analysis based on the sequence of the S gene. Sequence of the AKAV isolate strain was marked by triangles (▲). The nucleotide sequences of other AKAV strains used in this study were obtained from GenBank. Details for the origin of each strain are shown in Table 1.

The complete gene sequences used to construct the S gene phylogenetic tree were downloaded from GenBank (Table 1), and more information about constructing the M and L gene phylogenetic trees is provided (Supplementary Tables S1, S2). The results showed that the isolate of S fragment obtained from this outbreak was most closely related to the isolate GXLCH02 (S: KY381272, M: KY381278, L: KY381283) from *Rhizomys pruinosus* in China in 2016 (Figure 3). However, the isolates of M and L fragments were mostly closely related to the isolate NM/BS/1 from bovine in China and GXLCH04 from *Rhizomys pruinosus* in China, respectively (Supplementary materials). After recombination and adaptation analysis of the three fragments of AKAV FS202301, no virus recombination was found. The identity of amino acids of different AKAV strains was calculated (Supplementary materials). The results showed that the nucleotides of isolates from different countries and years had high consistency, indicating that AKAV has not undergone significant mutations.

**Table 1:** Detailed information on the AKAV of S gene used in the analysis.

S. No.	Virus strains	Country	Year	Accession number	Host	Sequence lengths
1	GXLCH70N	China	2016	KY381275	Rodent	complete cds
2	GXLCH16-70	China	2016	KY381274	Rodent	complete cds
3	GXLCH04	China	2016	KY381273	Rodent	complete cds
4	GXLCH01	China	2016	KY385908	Rodent	complete cds
5	GXLCH02	China	2016	KY381272	Rodent	complete cds
6	NM/BS/1	China	-	KU375444	Cattle	complete cds
7	DHL10M110	China	2010	KP144999	Mosquito	complete sequence
8	GXDH01	China	2016	MH174977	Goat	complete cds
9	YG-88-2	Japan	1988	AB232196	–	complete cds
10	AKAV-32/SKR/2010	South Korea	2010	JQ308773	Cattle	complete cds
11	AKAV-17/SKR/2010	South Korea	2010	JQ308772	Cattle	complete cds
12	Okayama2001	Japan	2001	AB289319	Cattle	complete sequence
13	KSB-3/P/06	Japan	2006	AB426280	Cattle	complete cds
14	KS-2/Mo/06	Japan	2006	AB373232	Cattle	complete cds
15	KM-2/Br/06	Japan	2006	AB426272	Cattle	complete sequence
16	Iriki	Japan	2001	AB289321	Cattle	complete sequence
17	CY-77	Taiwan	1993	AB232319	Cattle	complete cds
18	JaGAr39	Japan	1959	AB000852	Mosquito	complete cds
19	JaLAB39	Australia	1959	KR260714	Mosquito	complete sequence
20	TJ2016	China	2016	MT755621	Cattle	complete sequence
21	ON-89-2	Japan	1989	AB000867	Cattle	complete cds
22	KT3377	Japan	1977	AB000857	Cattle	complete cds
23	93FMX	South Korea	1993	FJ498797	Cattle	complete cds
24	OBE-1	Japan	1974	AB000851	–	complete sequence
25	AK7	South Korea	2006	FJ498795	Cattle	complete cds
26	TS-C2	Japan	–	AB968527	Cattle	complete sequence
27	K0505	South Korea	2005	FJ498796	Cattle	complete cds
28	R7949	Australia	1968	MH734959	Cattle	complete cds
29	B8935	Australia	1968	MH734940	Cattle	complete cds
30	MP496	Kenya	1972	AB232320	Mosquito	complete cds
31	KM-1/Br/06	Japan	2006	AB426271	Cattle	complete cds
32	AKAV FS202301	China	2023	PQ567128	Cattle	complete sequence

## Discussion

The Akabane disease is mainly distributed in tropical and temperate regions and has been reported in many countries, such as Japan, South Korea, China, Australia, and other countries (13, 18, 20–22), especially in Japan, where large-scale epidemics of the disease have occurred for many times (20, 40), causing huge economic losses for livestock breeding. Up to now, all the AKAV strains reported in Japan, South Korea, and China belong to genogroup I and genogroup II. Among them, the large-scale infection of AKAV in cattle and encephalomyelitis reported in South Korea in 2010 showed that its typical strain had the highest homology with the Japanese strain Iriki after isolation and identification, belonging to genogroup I (41). The increasingly frequent trade between countries can aggravate the spread of AKAV, but there is no effective drug against AKAV. Administering the AKAV vaccine prior to the active season of *Culicoides* is the optimal approach to prevent AKAV. The TS-C2 strain of Akabane virus (AKAV), attenuated by low-temperature adaptation and cell culture passages, demonstrated reduced pathogenicity and immunogenicity, showing potential as a live-attenuated vaccine candidate (42). Inactivated vaccines have been developed by Korean researchers (43, 44). Based on current research, potential Akabane virus (AKAV) vaccines could focus on subunit vaccines based on the Gc fragment and on attenuated viruses generated using reverse genetics to delete or partially delete the NSm protein, which may offer a safe and immunogenic approach to prevent AKAV infections (32, 45, 46).

In this study, a case of AKAV infection in Chinese cattle was reported, and the phylogenetic characteristics of AKAV based on the genetic sequence obtained from this outbreak were analyzed. The AKAV obtained in this study belonged to genogroup I and was closely related to the strain isolated from *Rhizomys pruinosus* in China. In the TEM analysis of Akabane virus (AKAV), spherical viral particles were observed, consistent with the size characteristics typical of members of the *Bunyaviridae* family. The phylogenetic information of AKAV strains reported in this study will enrich the epidemiological data of AKAV. More attention should be paid on AKAV, and more epidemiological investigations regarding AKAV should be conducted to obtain its epidemiological dynamics, thereby enabling the implementation of more effective measures for the prevention and control of Akabane disease.

## Data Availability

The datasets presented in this study can be found in online repositories. The names of the repository/repositories and accession number(s) can be found in the article/Supplementary material.

## Footnotes

^1^ https://megasoftware.net/
