# Correction: Isolation and identification of an AKAV strain in dairy cattle in China

**DOI:** 10.3389/fvets.2025.1641856

**Published:** 2025-06-25

**Authors:** Frontiers Production Office

**Affiliations:** Frontiers Media SA, Lausanne, Switzerland

**Keywords:** Akabane virus (AKAV), bovine abortion, molecular detection, phylogenetic analysis, transmission electron microscopy (TEM)

Author Keshan Zhang was erroneously not assigned as corresponding author.

The original version of this article has been updated.

