# Supplementary material for: Isolation and identification of an AKAV strain in dairy cattle in China
Source: Front Vet Sci. 2025 May 20;12:1574667. doi: 10.3389/fvets.2025.1574667 (PMC12131916; doi:10.3389/fvets.2025.1574667)
Supplement: Supplementary file 2 [file Table_2.docx]

Table S1. Detailed information on the AKAV of M gene used in the analysis

| **S.No.** | **Virus strains** | **Country** | **Year** | **Accession number** | **Host** | **Sequences lengths** |
| --- | --- | --- | --- | --- | --- | --- |
| 1 | GXLCH70N | China | 2016 | KY381281 | Rodent | complete cds |
| 2 | GXLCH16-70 | China | 2016 | KY381280 | Rodent | complete cds |
| 3 | GXLCH04 | China | 2016 | KY381279 | Rodent | complete cds |
| 4 | GXLCH01 | China | 2016 | KY381277 | Rodent | complete cds |
| 5 | GXLCH02 | China | 2016 | KY381278 | Rodent | complete cds |
| 6 | NM/BS/1 | China | - | KU375443 | Cattle | complete cds |
| 7 | DHL10M110 | China | 2010 | KY284022 | Mosquito | complete sequence |
| 8 | GXDH01 | China | 2016 | MH174978 | Goat | complete cds |
| 9 | YG-88-2 | Japan | 1988 | AB297827 | - | complete cds |
| 10 | AKAV-32/SKR/2010 | South Korea | 2010 | JQ308777 | Cattle | complete cds |
| 11 | AKAV-17/SKR/2010 | South Korea | 2010 | JQ308776 | Cattle | complete cds |
| 12 | Okayama2001 | Japan | 2001 | AB289322 | Cattle | complete sequence |
| 13 | KSB-3/P/06 | Japan | 2006 | AB426282 | Cattle | complete cds |
| 14 | KS-2/Mo/06 | Japan | 2006 | AB373233 | Cattle | complete cds |
| 15 | KM-2/Br/06 | Japan | 2006 | LC552048 | Cattle | complete sequence |
| 16 | Iriki | Japan | 2001 | AB297820 | Cattle | complete sequence |
| 17 | CY-77 | Taiwan | 1993 | AB297851 | Cattle | complete cds |
| 18 | JaGAr39 | Japan | 1959 | AB297818 | Mosquito | complete cds |
| 19 | JaLAB39 | Australia | 1959 | KR260715 | Mosquito | complete sequence |
| 20 | TJ2016 | China | 2016 | MT761688 | Cattle | complete sequence |
| 21 | ON-89-2 | Japan | 1989 | AB297828 | Cattle | complete cds |
| 22 | KT3377 | Japan | 1977 | AB297819 | Cattle | complete cds |
| 23 | 93FMX | South Korea | 1993 | FJ498799 | Cattle | complete cds |
| 24 | OBE-1 | Japan | 1974 | NC_009895 | - | complete sequence |
| 25 | AK7 | South Korea | 2006 | FJ498801 | Cattle | complete cds |
| 26 | TS-C2 | Japan | - | AB968526 | Cattle | complete sequence |
| 27 | K0505 | South Korea | 2005 | PQ799177 | Cattle | complete sequence |
| 28 | R7949 | Australia | 1968 | MH735016 | Cattle | complete cds |
| 29 | B8935 | Australia | 1968 | MH734997 | Cattle | complete cds |
| 30 | MP496 | Kenya | 1972 | AB297850 | Mosquito | complete cds |
| 31 | KM-1/Br/06 | Japan | 2006 | AB436954 | Cattle | complete cds |
| 32 | AKAV FS202301 | China | 2023 | PQ567127 | Cattle | complete sequence |

Table S2. Detailed information on the AKAV of L gene used in the analysis

| **S.No.** | **Virus strains** | **Country** | **Year** | **Accession number** | **Host** | **Sequences lengths** |
| --- | --- | --- | --- | --- | --- | --- |
| 1 | GXLCH70N | China | 2016 | KY381286 | Rodent | complete cds |
| 2 | GXLCH16-70 | China | 2016 | KY381285 | Rodent | complete cds |
| 3 | GXLCH04 | China | 2016 | KY381284 | Rodent | complete cds |
| 4 | GXLCH01 | China | 2016 | KY381282 | Rodent | complete cds |
| 5 | GXLCH02 | China | 2016 | KY381283 | Rodent | complete cds |
| 6 | GXDH01 | China | 2016 | MH174979 | Goat | complete cds |
| 7 | KM-2/Br/06 | Japan | 2006 | LC552049 | Cattle | complete sequence |
| 8 | JaLAB39 | Australia | 1959 | KR260716 | Mosquito | complete sequence |
| 9 | TJ2016 | China | 2016 | MT761689 | Cattle | complete sequence |
| 10 | OBE-1 | Japan | 1974 | NC_009894 | - | complete sequence |
| 11 | TS-C2 | Japan | - | AB968525 | Cattle | complete sequence |
| 12 | R7949 | Australia | 1968 | MH735073 | Cattle | complete cds |
| 13 | B8935 | Australia | 1968 | MH735054 | Cattle | complete cds |
| 14 | AKAV FS202301 | China | 2023 | PQ567126 | Cattle | complete sequence |
