# Supplementary material for: Isolation and identification of an AKAV strain in dairy cattle in China
Source: Front Vet Sci. 2025 May 20;12:1574667. doi: 10.3389/fvets.2025.1574667 (PMC12131916; doi:10.3389/fvets.2025.1574667)
Supplement: Supplementary file 3 [file Data_Sheet_1.pdf]

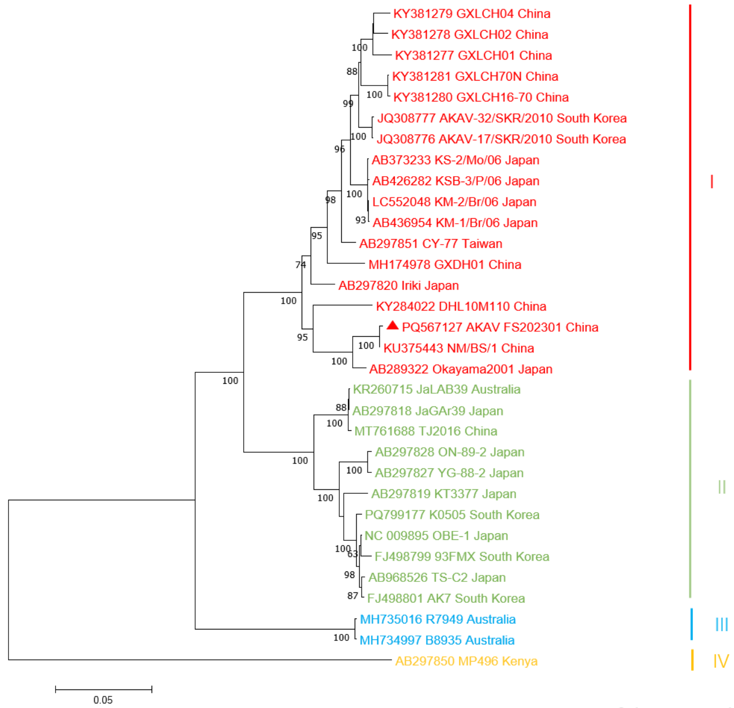

Phylogenetic analysis based on the sequence of the M gene. Sequence of the AKAV isolate strain was marked by triangles (▲). The nucleotide sequences of other AKAV strains used in this study were obtained from GenBank.

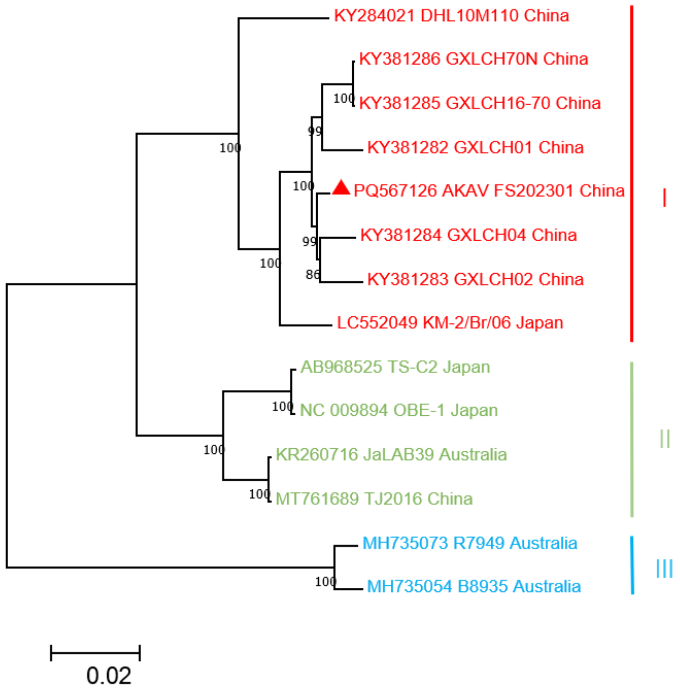

Phylogenetic analysis based on the sequence of the L gene. Sequence of the AKAV isolate strain was marked by triangles (▲). The nucleotide sequences of other AKAV strains used in this study were obtained from GenBank.
